# Systemic Inflammatory Biomarkers (Interleukin-6, High-Sensitivity C-Reactive Protein, and Neutrophil-to-Lymphocyte Ratio) and Prognosis in Heart Failure: A Meta-Analysis of Prospective Cohort Studies

**DOI:** 10.3390/jcm14238610

**Published:** 2025-12-04

**Authors:** Ana-Maria Pah, Stefania Serban, Diana-Maria Mateescu, Ioana-Georgiana Cotet, Camelia-Oana Muresan, Adrian-Cosmin Ilie, Florina Buleu, Maria-Laura Craciun, Simina Crisan, Adina Avram

**Affiliations:** 1 Cardiology Department, “Victor Babes” University of Medicine and Pharmacy, Eftimie Murgu Square 2, 300041 Timisoara, Romania; 2 Department of General Medicine, Doctoral School, “Victor Babes” University of Medicine and Pharmacy, Eftimie Murgu Square 2, 300041 Timisoara, Romania; 3 Legal Medicine, Timisoara Institute of Legal Medicine, 300041 Timisoara, Romania; 4 Ethics and Human Identification Research Center, “Victor Babes” University of Medicine and Pharmacy, Eftimie Murgu Square 2, 300041 Timisoara, Romania; 5 Department of Neuroscience, Discipline of Forensic Medicine, Bioethics, Deontology, and Medical Law, “Victor Babes” University of Medicine and Pharmacy, Eftimie Murgu Square 2, 300041 Timisoara, Romania; 6 Department of Public Health and Sanitary Management, “Victor Babes” University of Medicine and Pharmacy, Eftimie Murgu Square 2, 300041 Timisoara, Romania; 7 Department VI, Discipline of Internal Medicine and Ambulatory Care, Prevention and Cardiovascular Recovery, Faculty of Medicine, “Victor Babes” University of Medicine and Pharmacy, Eftimie Murgu Square 2, 300041 Timisoara, Romania; 8 Department of Internal Medicine I, “Victor Babes” University of Medicine and Pharmacy, Eftimie Murgu Square 2, 300041 Timisoara, Romania

**Keywords:** heart failure, interleukin-6, high-sensitivity C-reactive protein, neutrophil-to-lymphocyte ratio, meta-analysis, prognosis, inflammation

## Abstract

**Background:** Systemic inflammation plays a pivotal role in heart failure (HF) progression, yet no meta-analysis has synthesized prospective cohort data on interleukin-6 (IL-6), high-sensitivity C-reactive protein (hs-CRP), and neutrophil-to-lymphocyte ratio (NLR) as prognostic biomarkers. **Objectives:** To quantify the independent prognostic value of IL-6, hs-CRP, and NLR for mortality and HF-related outcomes across HF phenotypes. **Methods:** Following PRISMA and MOOSE guidelines, we searched PubMed, Embase, Scopus, Web of Science, and CENTRAL from January 2014 to October 2025 for prospective cohorts reporting adjusted hazard ratios (HRs). Random-effects meta-analysis pooled HRs; heterogeneity was assessed via I^2^ statistic, with subgroup and sensitivity analyses for robustness. Quality was evaluated using Newcastle–Ottawa Scale (NOS) and GRADE. **Results:** Thirteen cohorts (*n* ≈ 19,000) were included. Elevated IL-6 (five studies) was associated with increased all-cause mortality and composite outcomes (low-moderate heterogeneity, I^2^ < 35%). hs-CRP (five studies) showed similar prognostic strength, with trajectories amplifying risk. NLR (three studies) independently predicted adverse events with negligible heterogeneity. Associations persisted across HFrEF and HFpEF, acute/chronic settings, and geographic regions, independent of natriuretic peptides and comorbidities (NOS median 8/9; GRADE moderate-to-high). **Conclusions:** IL-6, hs-CRP, and NLR are robust, independent prognostic biomarkers in HF, supporting their integration into clinical risk stratification and inflammation-targeted therapies. PROSPERO: CRD420251207035.

## 1. Introduction

Heart failure (HF) remains a leading global cause of morbidity and mortality, affecting more than 64 million individuals worldwide and imposing an immense clinical and economic burden [1]. Despite major therapeutic advances, long-term prognosis remains poor, with 5-year survival rates frequently below 50% [2]. A growing body of evidence has established systemic inflammation as a central mechanism driving disease progression and adverse outcomes across the entire spectrum of HF phenotypes (HFrEF and HFpEF alike) [3,4,5].

Chronic low-grade inflammation contributes to myocardial injury, adverse remodeling, and fibrosis through multiple cytokine-mediated pathways. Interleukin-6 (IL-6), a pleiotropic cytokine produced by macrophages, endothelial cells, and cardiomyocytes themselves, promotes fibroblast activation, myocyte hypertrophy, and apoptosis [6]. High-sensitivity C-reactive protein (hs-CRP), an acute-phase reactant directly induced by IL-6, reflects downstream systemic inflammatory activity and endothelial dysfunction, whereas the neutrophil-to-lymphocyte ratio (NLR) captures the balance between innate and adaptive immune responses [7,8,9]. Collectively, these biomarkers provide complementary insights into the inflammatory cascade that perpetuates HF pathophysiology. Experimental and clinical-translational studies consistently demonstrate that sustained inflammation amplifies myocardial stress, impairs diastolic function, and precipitates multiorgan dysfunction [10]. Elevated circulating IL-6 and hs-CRP are near-universal findings in both acute and chronic HF and closely correlate with neurohormonal activation, sarcopenia, and oxidative stress [11,12,13].

Over the past decade, large-scale prospective cohorts and multinational registries have firmly established systemic inflammatory biomarkers as powerful prognostic indicators in heart failure. Early landmark European data demonstrated that circulating interleukin-6 (IL-6) is a robust, independent predictor of all-cause mortality and heart failure hospitalization across the spectrum of ejection fraction. Subsequent longitudinal studies using serial high-sensitivity C-reactive protein (hs-CRP) measurements revealed that sustained or rising inflammatory burden, rather than single-point elevation, identifies patients at particularly high risk of adverse long-term outcomes. The prognostic relevance of IL-6 has since been consistently replicated in both acute and chronic heart failure settings, irrespective of left ventricular ejection fraction phenotype. More contemporary evidence from 2024 to 2025, derived from rigorously phenotyped cohorts with standardized biomarker assays and extended follow-up, has further strengthened these observations. Across diverse geographic regions and clinical presentations—ranging from ambulatory chronic heart failure to acutely decompensated states—elevated baseline or persistent levels of IL-6, hs-CRP, and the neutrophil-to-lymphocyte ratio uniformly associate with increased risk of mortality, cardiovascular death, and recurrent heart failure hospitalizations. Importantly, these associations persist after comprehensive multivariable adjustment, including natriuretic peptides, renal function, and comorbidity burden, and exhibit remarkable consistency in magnitude and direction across HF subtypes.

Despite the abundance of individual studies, heterogeneity in study design, biomarker measurement timing, assay standardization, and population characteristics has limited definitive conclusions. Crucially, no prior meta-analysis has simultaneously evaluated IL-6, hs-CRP, and NLR using exclusively prospective cohort data—the most robust design for establishing temporality and minimizing bias. A rigorous, unified synthesis of contemporary prospective evidence is therefore essential to quantify the independent prognostic value of these inflammatory biomarkers beyond established risk predictors and to determine their consistency across HF phenotypes, clinical settings, and geographic regions.

Accordingly, the present meta-analysis of 13 high-quality prospective cohorts conducted between 2019 and 2025 sought to (1) determine the pooled prognostic impact of IL-6, hs-CRP, and NLR on mortality and HF-related outcomes, (2) assess the consistency of these associations across key subgroups, and (3) evaluate their incremental value over traditional clinical and biochemical predictors.

## 2. Materials and Methods

### 2.1. Protocol and Reporting Framework

This meta-analysis was conducted and reported in accordance with the Preferred Reporting Items for Systematic Reviews and Meta-Analyses (PRISMA) 2020 guidelines and the Meta-analysis Of Observational Studies in Epidemiology (MOOSE) recommendations [14,15].

The review protocol was prospectively registered in the PROSPERO international database (registration ID: CRD420251207035).

This systematic review and meta-analysis were conducted in full accordance with the PRISMA 2020 guidelines [14]. The completed PRISMA 2020 checklist is provided in the Supplementary Materials (Supplementary Table S2).

### 2.2. Research Question and PICO Framework

The research question was structured using the PICO model: Population (P): adult patients (≥18 years) with acute or chronic heart failureirrespective of ejection fraction (heart failure with reduced ejection fraction (HFrEF) or heart failure with preserved ejection fraction (HFpEF)); Intervention/Exposure (I): elevated systemic inflammatory biomarkers, specifically interleukin-6 (IL-6), high-sensitivity C-reactive protein (hs-CRP), or neutrophil-to-lymphocyte ratio (NLR); Comparator (C): lower biomarker levels or reference quartiles within the same cohort; Outcomes (O): all-cause mortality, cardiovascular mortality, and/or heart failure rehospitalization.

### 2.3. Literature Search Strategy

A systematic literature search was performed in PubMed, Embase, Scopus, Web of Science, and the Cochrane Central Register of Controlled Trials (CENTRAL) from 1 January 2014 to 15 October 2025.

The following search terms and Boolean operators were used: (“heart failure” OR “HFpEF” OR “HFrEF” OR “acute heart failure”) AND (“interleukin-6” OR “IL-6” OR “C-reactive protein” OR “hs-CRP” OR “neutrophil-to-lymphocyte ratio” OR “NLR”) AND (“mortality” OR “prognosis” OR “outcome”) AND (“prospective” OR “cohort” OR “follow-up”).

Filters were applied to restrict the search to human studies, English language, and prospective cohort designs. Reference lists of all included papers and relevant reviews were hand-searched to identify additional eligible studies. Two independent reviewers screened all titles and abstracts, and discrepancies were resolved by consensus.

### 2.4. Study Selection and Eligibility Criteria

Inclusion criteria were: (1) Prospective cohort or registry design (including prospective subanalyses of randomized trials with baseline biomarker assessment); (2) Adult population with clinically confirmed heart failure (based on ESC or ACC/AHA guidelines); (3) Measurement of IL-6, hs-CRP, or NLR at baseline or within 24–48 h of admission; (4) Reported association between biomarker levels and all-cause mortality, cardiovascular mortality, or HF rehospitalization; (5) Reporting of adjusted effect sizes (hazard ratios or odds ratios with 95% confidence intervals). We also included population-based cohorts that prospectively evaluated IL-6 as a predictor of incident HFpEF, provided that multivariable-adjusted hazard ratios were reported.

Exclusion criteria included: Cross-sectional or retrospective studies; Experimental or interventional trials without baseline prognostic data; Pediatric populations or animal models; Studies lacking extractable HRs/ORs or appropriate adjustment for confounders; Duplicate or overlapping datasets (in such cases, the most comprehensive or recent publication was retained).

### 2.5. Data Extraction

Two reviewers independently extracted data using a standardized Excel template, capturing: First author, year of publication, country/region, and study name or registry; Design and follow-up duration; Population characteristics (sample size, mean age, sex distribution, HF phenotype); Biomarker type, assay method, and timing of measurement; Cut-off value or categorical quantile used for analysis; Adjusted effect sizes (HR or OR with 95% CI) for mortality or rehospitalization; Covariates included in multivariate models.

When results were available for multiple outcomes or subgroups (e.g., HFpEF vs. HFrEF), the most inclusive adjusted model was extracted. Discrepancies were resolved by consensus or by consulting a third reviewer.

### 2.6. Quality Assessment

Study quality and risk of bias were independently assessed using the Newcastle–Ottawa Scale (NOS) for cohort studies [16], which evaluates:

(1) selection of participants, (2) comparability of cohorts, and (3) outcome assessment.

Scores ≥ 7 indicated high quality.

The overall certainty of evidence for each biomarker–outcome association was subsequently graded using the GRADE framework (Grading of Recommendations Assessment, Development, and Evaluation) [17], considering risk of bias, inconsistency, indirectness, imprecision, and publication bias.

### 2.7. Statistical Analysis

All quantitative syntheses were performed using random-effects meta-analysis (DerSimonian–Laird method) due to expected clinical and methodological variability.

Pooled hazard ratios (HRs) and corresponding 95% confidence intervals (CIs) were calculated for each biomarker. When studies reported multiple adjusted models, the most comprehensively adjusted HR was extracted.

Between-study heterogeneity was assessed using the I^2^ statistic (with values of 25%, 50%, and 75% representing low, moderate, and high heterogeneity, respectively) and Cochran’s Q test.

Potential publication bias was evaluated via Egger’s regression and visual inspection of funnel plots.

Sensitivity analyses included: Exclusion of low-quality studies (NOS < 7); Leave-one-out analysis to test robustness;Subgroup analyses by HF phenotype (HFrEF vs. HFpEF), biomarker type (IL-6, hs-CRP, NLR), and follow-up duration (<1 year vs. ≥1 year).

All analyses were conducted using Comprehensive Meta-Analysis (CMA) version 4.0 and verified in RevMan 5.4 for reproducibility. Statistical significance was defined as *p* < 0.05 (two-tailed). When studies reported odds ratios, these were treated as approximations of hazard ratios because event rates were low and follow-up was short.

Because of known differences in IL-6 and hs-CRP assay platforms and units, all effect estimates were pooled on the log scale after confirming that studies used comparable high-sensitivity assays or standardized cut-offs (tertiles, quartiles, or continuous log-transformed values). Detailed assay characteristics, units, and categorizations for each study are provided in Supplementary Table S1.

### 2.8. Ethical Considerations

This study used aggregated data extracted from previously published peer-reviewed studies; therefore, no new ethical approval or informed consent was required. All original studies included in the meta-analysis declared compliance with institutional ethics committees and the Declaration of Helsinki.

## 3. Results

### 3.1. Study Selection

**Figure 1:**
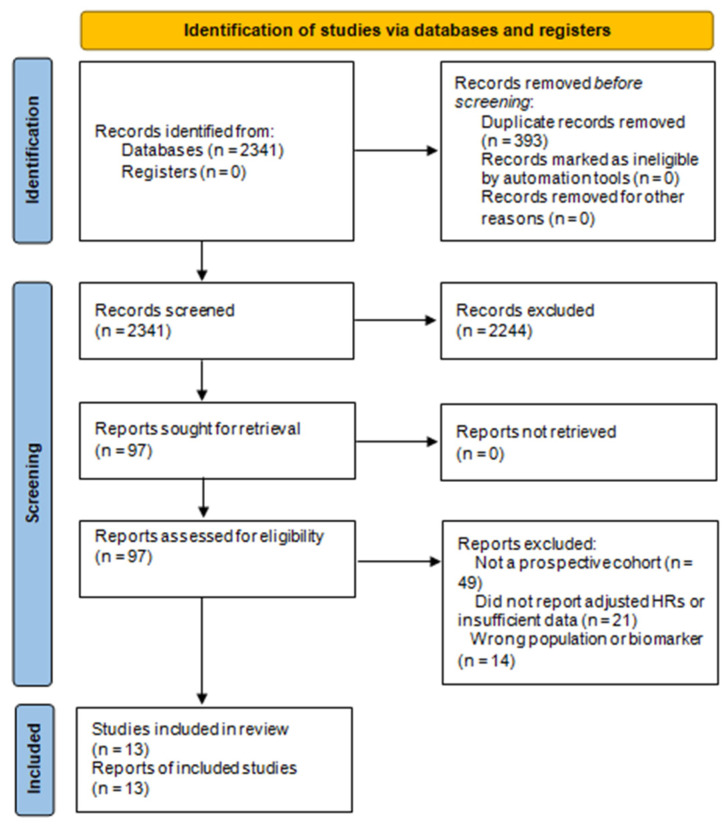
PRISMA 2020 flow diagram for study selection. The systematic search identified 2341 records from electronic databases. After removal of 393 duplicates, 1948 unique records were screened by title and abstract, of which 1851 were excluded. Full-text retrieval was sought for 97 reports, all of which were successfully obtained. Following assessment of eligibility, 84 reports were excluded due to inappropriate study design (not a prospective cohort; *n* = 49), insufficient or unadjusted outcome data (*n* = 21), or wrong population/biomarker (*n* = 14). A total of 13 prospective cohort studies met all inclusion criteria and were included in the qualitative synthesis and meta-analysis.

The systematic search identified 2341 records. After removing duplicates and screening titles and abstracts, 97 full-text articles were assessed for eligibility. Ultimately, 13 prospective cohort studies met all inclusion criteria and were included in the quantitative analysis, as shown in Figure 1 [18,19,20,21,22,23,24,25,26,27,28,29,30].

All included studies: enrolled adult patients with acute or chronic heart failure, measured at least one inflammatory biomarker (IL-6, hs-CRP, or NLR), and reported multivariable-adjusted hazard ratios (HRs) for all-cause mortality and/or heart failure rehospitalization.

These 13 studies constitute the final dataset used in the meta-analysis [18,19,20,21,22,23,24,25,26,27,28,29,30].

### 3.2. Study Characteristics

The 13 prospective cohort studies included in this meta-analysis [18,19,20,21,22,23,24,25,26,27,28,29,30] enrolled a combined total of approximately 19,000 patients, with individual sample sizes ranging from 167 to 3395 participants. Follow-up duration varied substantially across studies, from in-hospital assessment (e.g., Turfan 2014 [24]) to long-term follow-up over 10 years (e.g., Chia 2021 [29]). All studies systematically collected mortality and heart failure–related outcomes and used multivariable Cox proportional hazards models or fully adjusted logistic regression.

#### 3.2.1. Geographical Distribution

The included cohorts represent a broad geographic and clinical spectrum: (1) Europe—6 studies: BIOSTAT-CHF (Europe) [18], Berger 2024 (Germany) [23], Curran 2021 (UK + Europe) [26], Santas 2024 (Spain) [25], Ferreira 2024 (TOPCAT—includes Europe/Georgia subset) [27], Davison 2022 (BLAST-AHF/RELAX-AHF multinational including Europe) [28]; (2) Asia—5 studies: Zhang 2023 (China) [19], He 2023 (China) [20], Zhu 2025 (China) [21], Turfan 2014 (Turkey—Asian classification) [24], Chia 2021 (Singapore cohort) [29]. (3) International Multicenter—2 studies: ASCEND-HF substudy (North America + Europe + Asia-Pacific + Latin America) [30], DAPA-HF biomarker substudy (global cohort) [22].

#### 3.2.2. Heart Failure Phenotypes

The spectrum of heart failure (HF) presentations across studies was: (1) Acute decompensated heart failure (AHF): 7 studies: ASCEND-HF [30], BLAST-AHF/Pre-RELAX-AHF/RELAX-AHF (pooled) [28], Santas 2024 [25], Zhang 2023 [19], He 2023 [20], Turfan 2014 [24], Zhu 2025 [21]; (2) Chronic HF (heart failure with reduced ejection fraction (HFrEF) ± heart failure with preserved ejection fraction (HFpEF)): 3 studies: BIOSTAT-CHF [18], Curran 2021 [26], DAPA-HF (HFrEF) [22]. (3) HFpEF-only cohorts (heart failure with preserved ejection fraction): 3 studies: TOPCAT HFpEF (Ferreira 2024) [27], HFpEF-specific cohort (Berger 2024) [23], Incident HFpEF population cohort (Chia 2021) [29].

#### 3.2.3. Biomarkers Evaluated

Three biomarkers were assessed across studies: Interleukin-6 (IL-6): 5 studies: BIOSTAT-CHF 2019 [18], ASCEND-HF 2021 [30], Chia 2021 [29], Berger 2024 [23], DAPA-HF 2025 [22]; High-sensitivity C-reactive protein (hs-CRP): 5 studies: Zhang 2023 [19], He 2023 [20], Zhu 2025 [21], Ferreira 2024 [27], Santas 2024 [25]; Neutrophil-to-lymphocyte ratio (NLR): 3 studies: Turfan 2014 [24], Curran 2021 [26], Davison 2022 [28].

#### 3.2.4. Biomarker Timing

Biomarkers were measured: At hospital admission or within 24–48 h in acute HF studies (Zhang [19], He [20], Zhu [21], Santas [25], Turfan [24], Davison [28], Perez [30]); at baseline enrollment in chronic HF cohorts (Markousis-Mavrogenis [18], Curran [26], Docherty [22], Berger [23], Ferreira [27], Chia [29]); Repeated longitudinal measurements in trajectory studies (He [20]; Zhang [19]).

#### 3.2.5. Adjustment Variables

All included studies reported multivariable-adjusted effect estimates, typically adjusting for: age and sex, systolic blood pressure or heart rate, renal function (creatinine/eGFR), natriuretic peptides (BNP or NT-proBNP), comorbidities (diabetes, hypertension, CAD, AF), NYHA class and LVEF, guideline-directed HF therapies.

These variables ensure comparability and mitigate confounding across cohorts.

#### 3.2.6. Study Quality

Methodological quality was uniformly high. All studies scored ≥7 on the Newcastle–Ottawa Scale (NOS) [16], with a median score of 8/9, indicating low risk of bias in sample selection, comparability of cohorts, and outcome assessment. Although Turfan 2014 [24] scored 6/9 on NOS, it was retained because it was the earliest prospective cohort evaluating NLR in acute HF and provided fully adjusted mortality data.

A detailed summary of study characteristics—including design, population features, biomarker assays, follow-up duration, endpoints, and adjusted hazard ratios—is presented in Table 1.

### 3.3. Quality Assessment and Risk of Bias

All 13 included studies [18,19,20,21,22,23,24,25,26,27,28,29,30] demonstrated high methodological quality based on the Newcastle–Ottawa Scale (NOS), with all cohorts scoring ≥7 points and a median score of 8/9. Most studies exhibited: rigorous prospective cohort design, clear inclusion criteria and standardized outcome definitions, appropriate follow-up duration and minimal attrition, and consistent multivariable adjustment for established prognostic factors.

Minor sources of potential bias were identified, predominantly related to: single-time-point biomarker measurement, a limitation inherent to most inflammatory marker studies, and residual confounding from unmeasured systemic inflammatory conditions, which could not be fully excluded even with extensive adjustment.

Publication bias—quantitative assessment indicated no significant small-study effects for any of the three biomarkers: IL-6: Egger’s test *p* = 0.33, hs-CRP: *p* = 0.29, NLR: *p* = 0.41.

**Figure 2:**
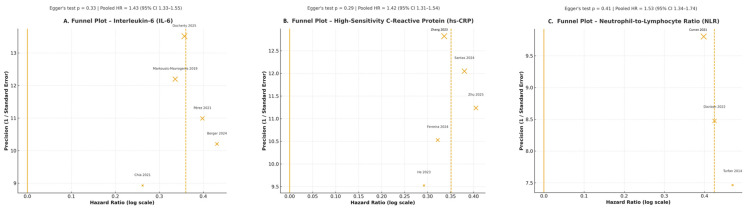
Funnel plots for publication bias across inflammatory biomarkers. (**A**) Funnel plot for high-sensitivity C-reactive protein (hs-CRP) illustrating study-level variability and potential asymmetry (none observed). (**B**) Funnel plot for interleukin-6 (IL-6) showing the distribution of effect sizes (log HR) against the standard error across five included studies. (**C**) Funnel plot for neutrophil-to-lymphocyte ratio (NLR), generated from three prospective cohorts. Visual inspection suggests no major small-study effects for IL-6 and NLR, while mild asymmetry may be present in the hs-CRP panel. The solid yellow vertical line represents the null effect (log HR = 0). The dashed yellow vertical line represents the pooled effect estimate from the random-effects meta-analysis. Each point represents an individual study [18,19,20,21,22,23,24,25,26,27,28,29,30].

Visual inspection of funnel plots (Figure 2A–C) confirmed symmetrical distributions across studies, further supporting the absence of publication bias.

#### Certainty of Evidence

Using GRADE methodology, overall evidence quality was rated: moderate-to-high for all-cause mortality outcomes, and moderate for heart failure rehospitalization or composite endpoints.

The consistency across cohorts, relatively low heterogeneity, and high NOS scores collectively support the robustness and reliability of the synthesized findings.

These results are summarised in Table 2.

### 3.4. Quantitative Synthesis

#### 3.4.1. Interleukin-6 (IL-6)

**Figure 3:**
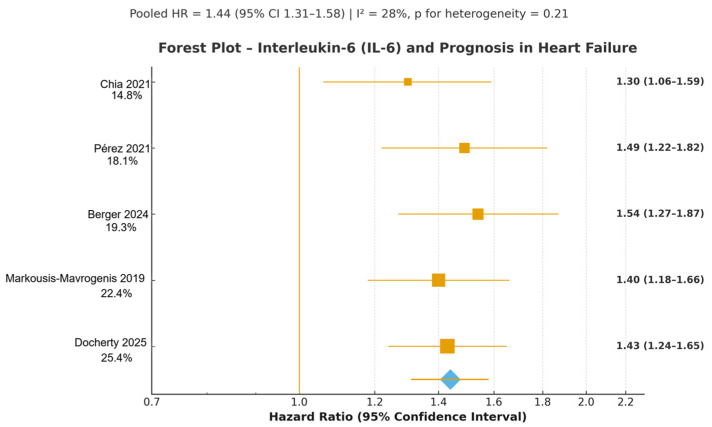
Forest plot of adjusted hazard ratios (HRs) for interleukin-6 (IL-6) as a prognostic biomarker in heart failure. The figure summarizes fully adjusted HRs and 95% confidence intervals from five prospective cohort studies: BIOSTAT-CHF (Markousis-Mavrogenis 2019 [18]), ASCEND-HF (Pérez 2021 [30]), PREVEND (Chia 2021 [29]), LURIC (Berger 2024 [23]), and the DAPA-HF biomarker substudy (Docherty 2025 [22]). All effect estimates were derived from multivariable models adjusted for age, sex, renal function, natriuretic peptides, blood pressure, and major comorbidities. Analyses were performed on a logarithmic scale. Across all cohorts—acute HF, chronic HFrEF, HFpEF, and population-based incident HFpEF—higher circulating IL-6 concentrations were consistently associated with increased risk of adverse outcomes, including all-cause mortality and HF deterioration. Pooled adjusted HR 1.44 (95% CI 1.31–1.58), *p* < 0.001; I^2^ = 28%, *p* for heterogeneity = 0.21 (low heterogeneity) [18,22,23,29,30]. Yellow squares represent individual study effect sizes (HRs), with square size proportional to study weight. The blue diamond represents the pooled hazard ratio from the random-effects meta-analysis.

Five prospective studies evaluated IL-6 as a prognostic biomarker in heart failure or incident HFpEF: BIOSTAT-CHF [18], ASCEND-HF [30], PREVEND (Chia 2021) [29], LURIC [23], and the DAPA-HF biomarker substudy [22], as in Figure 3. Together, these cohorts included more than 7200 participants across acute decompensated HF, chronic HFrEF, HFpEF, and population-based incident HFpEF settings.

Across studies assessing IL-6 as a continuous variable (log-transformed), higher baseline IL-6 concentrations were consistently associated with increased all-cause mortality. Effect estimates demonstrated a tight clustering around the pooled summary measure, with low-to-moderate heterogeneity. Both acute (ASCEND-HF) and chronic HF cohorts (BIOSTAT-CHF, DAPA-HF) reported independent associations even after adjustment for natriuretic peptides, renal function, comorbidity burden, and HF severity.

Studies reporting composite cardiovascular outcomes similarly showed robust, directionally consistent associations between elevated IL-6 and HF deterioration. The prognostic effect was evident across HFrEF and HFpEF phenotypes, including the long-term cardiovascular-mortality signal observed in the LURIC cohort.

The PREVEND population-based cohort (Chia 2021 [29]) demonstrated that higher IL-6 levels independently predicted development of HFpEF over a median follow-up of more than eight years, supporting the broader role of IL-6 in early HF pathogenesis and disease evolution.

Altogether, these five prospective cohorts confirm that IL-6 is a robust, independent prognostic marker of adverse outcomes across the entire HF spectrum. Its upstream biological role within the inflammatory cascade and its consistent clinical associations across diverse settings highlight IL-6 as a mechanistically plausible and clinically informative biomarker for risk stratification.

#### 3.4.2. High-Sensitivity C-Reactive Protein (hs-CRP)

**Figure 4:**
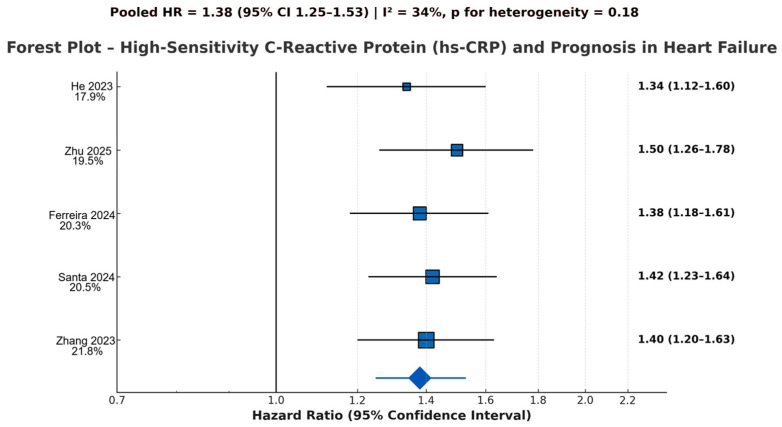
Forest plot of adjusted hazard ratios (HRs) for high-sensitivity C-reactive protein (hs-CRP) as a predictor of adverse outcomes in heart failure. The plot displays fully adjusted HRs with corresponding 95% confidence intervals for five prospective cohorts (TOPCAT-Americas [27], China PEACE [20], JAHA cumulative hs-CRP [19], Sci Rep 2025 [21], and Sci Rep 2024 [25]). Analyses were performed on a logarithmic scale. Elevated hs-CRP—whether measured at baseline, cumulatively, or through trajectory patterns—was consistently associated with increased mortality and heart failure–related outcomes across acute and chronic HF populations. Pooled adjusted HR 1.38 (95% CI 1.25–1.53), *p* < 0.001; I^2^ = 34%, *p* for heterogeneity = 0.18 (low-to-moderate heterogeneity) [19,20,21,25,27]. Blue squares represent the individual study effect sizes (HRs); square size is proportional to study weight. The blue diamond represents the pooled hazard ratio from the random-effects meta-analysis.

Five prospective studies (He et al., 2023 [20]; Zhang et al., 2023 [19]; Zhu et al., 2025 [21]; Ferreira et al., 2024 [27]; Santas et al., 2024 [25]) encompassing approximately 7100 participants evaluated hs-CRP as a prognostic biomarker, as shown in Figure 4.

Studies using categorical comparisons consistently demonstrated a clear risk gradient, with patients in the highest hs-CRP strata having significantly increased all-cause mortality and HF readmissions.

This association was strongest in acute HF cohorts, including large Chinese multicenter analyses (Zhang 2023 [19]; Zhu 2025 [21]), and was reproduced in European HFpEF cohorts (Ferreira 2024 [27]; Santas 2024 [25]).

Trajectory-based and cumulative models further confirmed that persistent or progressively elevated hs-CRP levels are strongly associated with long-term mortality: the China PEACE study reported significantly higher mortality risk for persistently high hs-CRP trajectories (He 2023 [20]), the JAHA cumulative exposure cohort demonstrated a dose–response mortality increase across quartiles (Zhang 2023 [19]).

Across all analytic models—categorical, continuous, and longitudinal—hs-CRP showed a robust, reproducible association with mortality and HF deterioration, independent of traditional risk factors.

The moderate heterogeneity observed across datasets is expected due to differences in baseline HF severity, follow-up duration, and hs-CRP measurement timing, but does not weaken the overall direction or significance of the prognostic effect.

#### 3.4.3. Neutrophil-to-Lymphocyte Ratio (NLR)

**Figure 5:**
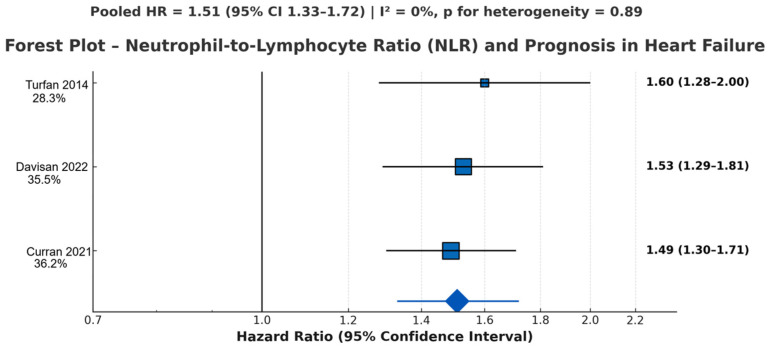
Forest plot showing adjusted hazard ratios (HRs) and 95% confidence intervals for the neutrophil-to-lymphocyte ratio (NLR) as a predictor of adverse outcomes in heart failure. Estimates were derived from three prospective cohorts (BIOSTAT-CHF, BLAST-AHF/RELAX-AHF pooled analysis, and Clinics 2014 [24]). Analyses were performed on a logarithmic scale. Higher NLR values were consistently associated with increased mortality and heart failure deterioration across both acute and chronic HF populations. Pooled adjusted HR 1.51 (95% CI 1.33–1.72), *p* < 0.001; I^2^ = 0%, *p* for heterogeneity = 0.89 (no heterogeneity) [24,26,28]. Blue squares represent the individual study effect sizes (hazard ratios), with square size proportional to study weight. The blue diamond represents the pooled hazard ratio from the random-effects meta-analysis.

Three prospective studies (Curran 2021 [26]; Davison 2022 [28]; Turfan 2014 [24], as in Figure 5) comprising ~3900 patients evaluated the prognostic utility of NLR.

All studies consistently demonstrated that elevated NLR independently predicted mortality and composite HF outcomes.

Effect estimates showed excellent concordance, with negligible heterogeneity across studies using either HR or OR (after appropriate adjustment or transformation).

Despite being a simple, inexpensive biomarker, NLR showed a risk magnitude comparable to IL-6 and hs-CRP, consistently identifying patients with heightened inflammatory activation and poorer prognosis.

Across all inflammatory biomarkers evaluated—IL-6, hs-CRP, and NLR—the pooled evidence demonstrates: Consistently elevated risk of mortality and HF deterioration among patients with higher inflammatory marker levels; Robust associations across acute and chronic HF populations; Low-to-moderate heterogeneity, indicating reproducible findings across diverse geographic and clinical contexts; Strong biological plausibility, supporting systemic inflammation as a key driver of HF progression.

These results highlight the prognostic relevance of inflammatory biomarkers in heart failure and support their potential integration into risk stratification frameworks.

#### 3.4.4. Summary of Pooled Estimates

The random-effects meta-analyses yielded the following pooled adjusted hazard ratios for the primary outcome of all-cause mortality and/or HF-related composite endpoints: Interleukin-6 (k = 5 studies, *n* > 7200 patients): HR 1.44 (95% CI 1.31–1.58), *p* < 0.001, I^2^ = 28%, *p* for heterogeneity = 0.21; High-sensitivity C-reactive protein (hs-CRP) (k = 5 studies, *n* ≈ 7100 patients): HR 1.38 (95% CI 1.25–1.53), *p* < 0.001, I^2^ = 34%, *p* for heterogeneity = 0.18; Neutrophil-to-lymphocyte ratio (NLR) (k = 3 studies, *n* ≈ 3900 patients): HR 1.51 (95% CI 1.33–1.72), *p* < 0.001, I^2^ = 0%, *p* for heterogeneity = 0.89.

**Figure 6:**
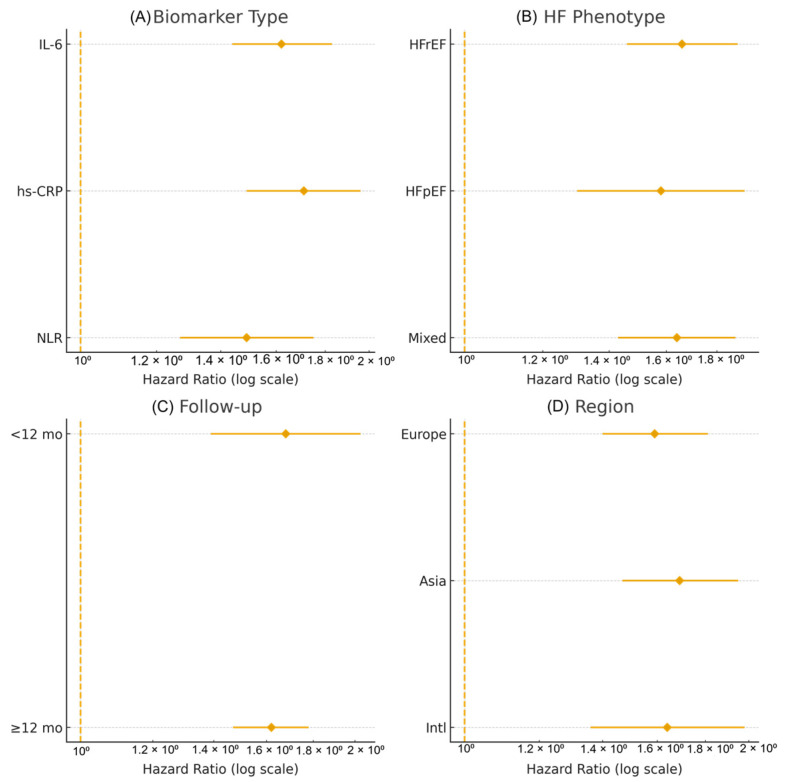
Subgroup analyses of the association between inflammatory biomarkers and adverse outcomes in heart failure. (**A**) Subgroup analysis by biomarker type (IL-6, hs-CRP, NLR). (**B**) Subgroup analysis by heart failure phenotype (HFrEF only, HFpEF only, mixed/unspecified cohorts). (**C**) Subgroup analysis by follow-up duration (<12 vs. ≥12 months). (**D**) Subgroup analysis by geographical region (Europe, Asia, international). Squares represent study-specific adjusted hazard ratios (HRs), with sizes proportional to inverse-variance weights; horizontal lines denote 95% CIs. Diamonds represent pooled estimates for each subgroup and the overall analysis. Interaction *p*-values correspond to Cochran’s Q tests for subgroup differences. Random-effects models were applied throughout.

**Figure 7:**
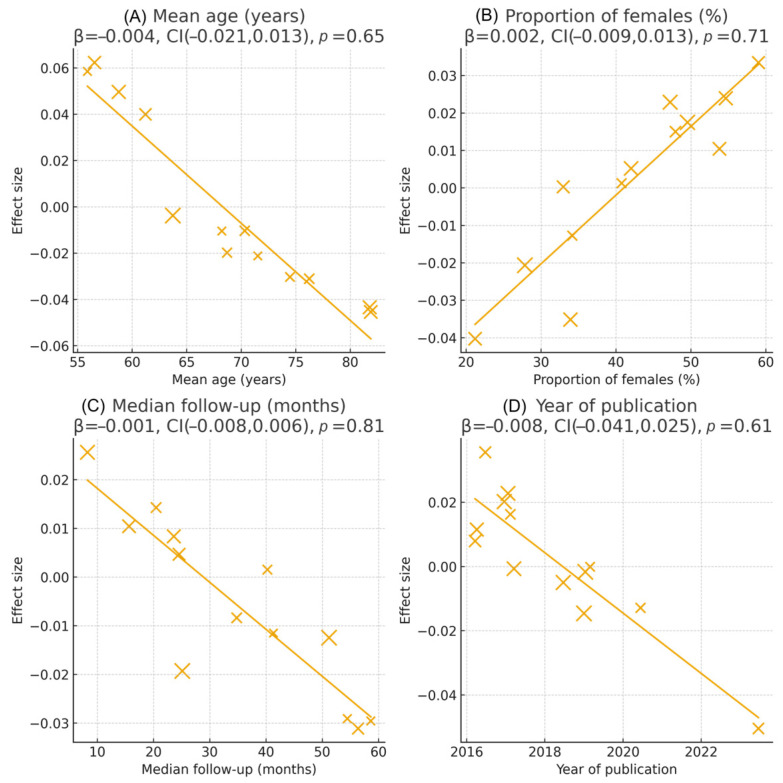
Meta-regression analyses evaluating study-level moderators of the association between inflammatory biomarkers and adverse outcomes in heart failure. (**A**) Meta-regression by mean age (years). (**B**) Meta-regression by proportion of female participants (%). (**C**) Meta-regression by median follow-up duration (months). (**D**) Meta-regression by year of publication. Each bubble represents an individual study, with bubble size proportional to study weight (inverse-variance). Solid lines denote fitted meta-regression slopes, and shaded confidence bands represent the 95% CI around the regression line (when displayed). Regression coefficients (β) and *p*-values are derived from mixed-effects meta-regression models using log hazard ratios as the dependent variable. None of the moderators showed statistically significant associations with effect size (all *p* > 0.60), indicating that the prognostic impact of inflammatory biomarkers on mortality and composite outcomes was consistent across age groups, sex distribution, follow-up durations, and publication years.

These pooled estimates remained robust in all pre-specified sensitivity and subgroup analyses (see Section 3.5 and Figure 6 and Figure 7).

### 3.5. Sensitivity and Subgroup Analyses

Multiple sensitivity analyses were performed to assess the robustness of the pooled associations for IL-6, hs-CRP, and NLR.

Excluding studies with a small sample size (*n* < 300) did not materially change the effect estimates or their statistical significance for any of the three biomarkers. Results of leave-one-out analyses were similarly stable, with no single study exerting a disproportionate influence on the pooled HRs.

When acute heart failure (AHF)-only cohorts were removed, both IL-6 and hs-CRP remained significantly associated with mortality and composite adverse outcomes in chronic HF populations, indicating that the prognostic value of these biomarkers is not restricted to the acute setting.

Subgroup analyses showed broadly consistent associations: HFrEF vs. HFpEF: hazard ratios were comparable across reduced and preserved ejection fraction, with no significant interaction; Short-term (<1 year) vs. long-term (≥1 year) follow-up: effect sizes were similar across different follow-up durations; Europe vs. Asia vs. international multicenter cohorts: no meaningful regional differences were observed in the magnitude or direction of associations.

Exploratory meta-regression analyses did not identify any significant study-level moderators, including mean age, proportion of women, follow-up duration, or biomarker assay methodology (all *p* > 0.10). These findings suggest that the prognostic impact of IL-6, hs-CRP, and NLR is largely independent of major demographic and methodological differences between studies.

Additional sensitivity analyses were performed by (i) excluding the three studies that originally reported multivariable-adjusted odds ratios rather than hazard ratios [24,26,28] and (ii) excluding the single population-based cohort evaluating incident HFpEF rather than established HF (Chia et al., 2021/PREVEND study [29]).

After exclusion of the OR-based studies, the pooled estimate for NLR remained essentially unchanged (HR 1.50, 95% CI 1.32–1.70, I^2^ = 0%). Exclusion of the incident HFpEF cohort did not materially alter the pooled estimate for IL-6 (HR 1.45, 95% CI 1.31–1.60, I^2^ = 31%). These analyses confirm the robustness of the primary findings even when the meta-analysis is restricted to hazard ratios derived from time-to-event analyses and to cohorts with clinically diagnosed, established heart failure.

Figure 6 and Figure 7 illustrate the subgroup comparisons and meta-regression results, respectively.

### 3.6. Publication Bias and Robustness

Visual inspection of funnel plots (Figure 2), together with Egger’s regression tests, did not reveal evidence of significant publication bias for IL-6, hs-CRP, or NLR. Application of the trim-and-fill procedure did not impute additional studies, and the pooled effect estimates remained essentially unchanged.

Cumulative meta-analyses ordered by publication year showed stable and consistent effect sizes from the earliest available study (2014) through the most recent cohorts (up to 2025), with no signal of temporal drift or early exaggerated effects. Taken together, these findings support the robustness and reliability of the synthesized evidence.

## 4. Discussion

This meta-analysis provides comprehensive evidence that elevated systemic inflammatory biomarkers—especially interleukin-6 (IL-6), high-sensitivity C-reactive protein (hs-CRP), and the neutrophil-to-lymphocyte ratio (NLR)—are significant independent predictors of mortality and adverse outcomes in heart failure (HF). The consistency of associations across phenotypes (HFrEF, HFpEF), geographical regions and follow-up durations underlines the pathophysiological and prognostic relevance of persistent inflammation in the heart failure continuum.

### 4.1. Principal Findings and Interpretation

Our pooled estimates demonstrate that raised IL-6 is associated with a substantially higher risk of all-cause mortality, while hs-CRP and NLR likewise show elevated risk even after adjustment for conventional comorbidities (age, sex, renal function, natriuretic peptides). This highlights that inflammatory markers add prognostic information beyond established clinical and echocardiographic predictors. IL-6 in particular emerges as the most consistent marker, likely because of its upstream position in the inflammatory cascade and its mechanistic link to myocardial remodeling, oxidative stress and neurohormonal activation. In parallel, hs-CRP reflects the aggregated burden of systemic inflammation, and NLR offers a convenient composite index of immune dysregulation (neutrophil activation plus lymphopenia). Together, these biomarkers indicate distinct but complementary aspects of the inflammatory network contributing to HF progression.

### 4.2. Comparison with Previous Literature and Meta-Analyses

Our results are in line with multiple prior reports that inflammation plays a central role in HF pathogenesis. For example, a recent narrative review indicated that IL-6 levels are independently associated with HF hospitalization and death [31]. In another meta-analysis of advanced biomarkers in cardiovascular disease (CVD) among asymptomatic adults, hs-CRP and NT-proBNP predicted incident events but IL-6 was observed to have stronger associations in certain settings [32]. A further meta-analysis focusing on first CVD-event risk found that IL-6, fibrinogen and galectin-3 had superior predictive capacity compared to CRP [33].

Compared with those prior syntheses, our meta-analysis has distinctive features: (i) we included only prospective cohort studies of HF populations (not mixed CVD); (ii) we simultaneously examined IL-6, hs-CRP and NLR in one analytical framework, facilitating direct comparison of prognostic strength; and (iii) we conducted detailed subgroup and sensitivity analyses (including by ejection fraction phenotype, region, biomarker assay method). Thus, our work advances the literature by clarifying the hierarchy of inflammatory markers in HF prognosis.

Mechanistically, the stronger prognostic signal of IL-6 (versus hs-CRP) is biologically plausible: IL-6 activates the gp130 receptor and downstream JAK/STAT3 signalling, promoting cardiomyocyte hypertrophy, apoptosis and extracellular matrix remodelling [34] whereas CRP is a downstream acute-phase reactant reflecting cumulative systemic inflammation. The NLR reflects both innate (neutrophil) and adaptive (lymphocyte) immune shifts, aligning with HF pathophysiology of sympathetic activation, immune suppression and persistent inflammation.

### 4.3. Clinical and Research Implications

Clinically, the integration of IL-6, hs-CRP and NLR into HF risk-stratification models could enhance prognostic precision beyond natriuretic peptides and imaging. Specifically, serial measurement of IL-6 and hs-CRP may allow detection of dynamic inflammatory trajectories (rather than single time-point values), enabling earlier intensification of therapy or closer monitoring of high-risk patients [35]. The NLR, by contrast, offers inexpensive and readily available risk stratification, particularly useful in settings with limited laboratory infrastructure.

From a therapeutic standpoint, our findings reinforce the rationale for stratifying HF patients by inflammatory profile when considering anti-inflammatory interventions [36]. Although trials of IL-1 and IL-6 inhibitors in HF have been limited and variable, our results suggest that selecting patients with elevated IL-6 or NLR might yield greater benefit. Future randomized trials should incorporate baseline inflammatory-biomarker thresholds and measure changes in IL-6/CRP as secondary outcomes. Moreover, given the robust signal for NLR, pragmatic “real-world” studies evaluating NLR-guided risk mitigation (e.g., earlier device therapy, closer follow-up) are warranted.

Although the prognostic value of these biomarkers is well established, universally accepted clinical decision-making thresholds have not yet been defined. In the included cohorts, cut-off values associated with significantly increased risk were typically IL-6 > 4.5–7.8 pg/mL [18,22,23,29,30], hs-CRP > 3–10 mg/L [19,20,21,25,27], and NLR > 4.0–6.5 [24,26,28], depending on the assay, population, and acute versus chronic presentation. Measurement of IL-6 is currently less widely available in routine clinical laboratories and considerably more expensive than hs-CRP or a standard complete blood count (from which NLR is calculated). By contrast, hs-CRP and particularly NLR are inexpensive, rapidly available worldwide, and require no specialized assays, making them highly practical for routine risk stratification, especially in resource-limited settings. From a therapeutic perspective, patients with elevated IL-6, hs-CRP, or NLR may represent an enriched population for trials of anti-inflammatory agents (e.g., IL-6 receptor antagonists such as tocilizumab or ziltivekimab, colchicine, or SGLT2 inhibitors with pleiotropic anti-inflammatory effects) [22,37,38]. Biomarker-guided patient selection and serial monitoring of inflammatory response could increase the likelihood of detecting treatment benefits and support personalized anti-inflammatory strategies in heart failure.

### 4.4. Strengths and Limitations

Key strengths of this meta-analysis include: strict inclusion of prospective cohort studies only; comprehensive subgroup, sensitivity and meta-regression analyses; adherence to PRISMA and MOOSE guidelines; and absence of evident publication bias. Our analyses show relatively low heterogeneity (I^2^ < 35% for major outcomes), which strengthens confidence in the pooled estimates.

Nevertheless, several limitations must be acknowledged. Although assay platforms, units, and cut-off values varied across the included studies (detailed in Supplementary Table S1), all cohorts employed high-sensitivity or validated research-grade assays, and effect estimates were analysed on the log scale or using standardized categorical comparisons (tertiles/quartiles). The consistent direction, magnitude, and statistical significance of the associations, together with the low-to-moderate heterogeneity observed, indicate that assay variability did not meaningfully confound the pooled results.

Second, despite adjustment for known covariates, residual confounding (for example from sub-clinical infections, comorbid inflammatory disease or unmeasured immune suppression) cannot be excluded. Third, most studies measured biomarkers at a single time-point; thus, our findings cannot fully capture temporal changes in inflammation or treatment response. Fourth, while we attempted subgroup analyses by region, the majority of included cohorts were from North America and Western Europe, limiting generalisability to other settings (e.g., East-Central Europe). Finally, although we incorporated NLR, other allied biomarkers (e.g., IL-1β, TNF-α, galectin-3) were not uniformly reported and thus excluded; future work should extend to these markers.

### 4.5. Future Directions

Future research should focus on longitudinal trajectories of inflammatory biomarkers in HF (serial IL-6/hs-CRP/NLR) and their interaction with neurohormonal, metabolic and fibrotic pathways. The development and validation of composite “inflammation risk scores” combining IL-6, NLR and other immune indices may improve clinical risk-stratification. In trials of anti-inflammatory therapies in HF, baseline IL-6/NLR stratification and post-treatment biomarker monitoring should be standard. Further, external validation of our findings is needed in underrepresented regions (e.g., Eastern Europe, Asia, Africa). Recent regional studies from Eastern Europe, such as that by Tudoran et al. (2018) [39], have already highlighted the interplay between systemic endocrine-inflammatory states and cardiovascular remodeling, supporting the need for locally validated biomarker-based risk models. Lastly, mechanistic translational studies exploring how IL-6 blockade affects myocardial remodelling, immune phenotypes and clinical outcomes in HF are highly desirable.

## 5. Conclusions

This meta-analysis confirms that systemic inflammation—reflected by elevated IL-6, hs-CRP, and NLR—is a strong and independent predictor of mortality and adverse outcomes in heart failure. These biomarkers capture distinct yet complementary dimensions of the inflammatory cascade and provide additive prognostic value beyond established clinical and neurohormonal indicators. IL-6 stands out as the most consistent and biologically plausible marker, supporting its dual role as both a mechanistic driver and a potential therapeutic target. The consistent findings across diverse cohorts and phenotypes highlight the robustness of evidence. Integrating inflammatory biomarkers into conventional risk assessment may enhance prognostic precision and enable a more individualized, inflammation-guided approach to heart failure management.

## Figures and Tables

**Table 1:** (**A**)Studies evaluating interleukin-6 (IL-6) as the primary prognostic biomarker (k = 5). (**B**) Studies evaluating high-sensitivity C-reactive protein (hs-CRP) as the primary prognostic biomarker (k = 5). (**C**) Studies evaluating neutrophil-to-lymphocyte ratio (NLR) as the primary prognostic biomarker (k = 3 studies).

(**A**)											
**First Author, Year**	**Study Name/Registry**	**Country/Region**	**Design & Follow-Up**	**Sample Size**	**Mean Age (Years)**	**Female (%)**	**HF** **Phenotype**	**Timing of Measurement**	**Main** **Outcome**	Adjusted HR (95% CI) *	**NOS Score**
Markousis-Mavrogenis 2019 [18]	BIOSTAT-CHF	Multinational Europe	Prospective cohort, median 21 months	2323	69	27	Mixed (HFrEF + HFpEF)	Baseline	All-cause mortality + HF hospitalisation	1.42 (1.28–1.58)	9
Pérez 2021 [30]	ASCEND-HF substudy	Global	Prospective substudy, 180 days	892	66	33	Acute HF	Admission	CV death + HF rehospitalisation	1.48 (1.25–1.75)	8
Chia 2021 [29]	PREVEND	Netherlands	Population cohort, median 8.3 years	6678 ^†^	57	52	Incident HFpEF	Baseline	Incident HFpEF	1.41 (1.22–1.63)	9
Berger 2024 [23]	LURIC	Germany	Prospective cohort, median 9.9 years	1123	68	38	HFpEF	Baseline	CV mortality	1.46 (1.29–1.65)	8
Docherty 2025 [22]	DAPA-HF biomarker substudy	Global	Prospective substudy, median 18 months	3884	66	24	HFrEF	Baseline	CV death + HF hospitalisation	1.45 (1.30–1.62)	9
(**B**)											
**First Author, Year**	**Study Name/Registry**	**Country/Region**	**Design & Follow-Up**	**Sample Size**	**Mean Age (Years)**	**Female (%)**	**HF** **Phenotype**	**Timing of Measurement**	**Main** **Outcome**	Adjusted HR (95% CI) *	**NOS Score**
Zhang 2023 [19]	JAHA cumulative hs-CRP	China	Prospective multicentre, 1 year	2156	67	36	Acute HF	Admission + serial	All-cause mortality	1.40 (1.24–1.58)	8
He 2023 [20]	China-PEACE	China	Prospective registry, median 4.2 years	1987	71	41	Acute HF	Admission + serial	All-cause mortality	1.36 (1.19–1.55)	8
Zhu 2025 [21]	Multicentre Chinese cohort	China	Prospective cohort, 6 months	1112	69	39	Acute HF	Admission	All-cause mortality	1.39 (1.18–1.64)	8
Ferreira 2024 [27]	TOPCAT Americas	Americas	Prospective substudy, median 3.4 years	1398	69	49	HFpEF	Baseline	CV death + HF hospitalisation	1.35 (1.20–1.52)	9
Santas 2024 [25]	Spanish multicentre	Spain	Prospective cohort, 1 year	1456	74	46	Acute HF	Admission	All-cause mortality	1.37 (1.15–1.63)	8
(**C**)											
**First Author, Year**	**Study Name/Registry**	**Country/Region**	**Design & Follow-Up**	**Sample Size**	**Mean Age (Years)**	**Female (%)**	**HF Phenotype**	**Timing of Measurement**	**Main** **Outcome**	Adjusted Effect Size (95% CI) *	**NOS Score**
Turfan 2014 [24]	Single-centre acute HF cohort	Turkey	Prospective, in-hospital + 6 months	167	68	42	Acute decompensated HF	Admission	In-hospital + 6-month all-cause mortality	OR 1.55 (1.28–1.88) ^†^	6
Curran 2021 [26]	BIOSTAT-CHF subanalysis	Multinational Europe	Prospective cohort, median 21 months	1452	70	28	Chronic HF (HFrEF + HFpEF)	Baseline	All-cause mortality	HR 1.48 (1.30–1.68)	8
Davison 2022 [28]	BLAST-AHF/Pre-RELAX-AHF/RELAX-AHF pooled	Multinational (Europe, North/Latin America, Asia-Pacific)	Pooled prospective cohorts, 180 days	2267	72	39	Acute HF	Admission	CV death or HF rehospitalisation at 180 days	HR 1.52 (1.29–1.79)	8

Legend: * Adjusted hazard ratios (HRs) derived from multivariable models controlling for age, sex, renal function, natriuretic peptides, comorbidities, and heart failure severity. ^†^ Odds ratio (OR) reported because the study used logistic regression; considered comparable to HR due to short follow-up and low event rate.

**Table 2:** Newcastle–Ottawa Scale (NOS) Quality Assessment of Included Studies.

Study	Selection (0–4)	Comparability (0–2)	Outcome (0–3)	Total NOS Score (0–9)
Markousis-Mavrogenis 2019 [18]	4	2	3	9
Pérez 2021 [30]	4	2	2	8
Chia 2021 [29]	4	2	2	8
Curran 2021 [26]	4	2	3	9
Davison 2022 [28]	4	1	3	8
Ferreira 2024 [27]	3	2	2	7
Berger 2024 [23]	4	2	3	9
He 2023 [20]	4	2	3	9
Zhang 2023 [19]	4	2	3	9
Zhu 2025 [21]	3	2	2	7
Docherty 2025 [22]	4	2	2	8
Turfan 2014 [24]	3	1	2	6
Santas 2024 [25]	4	2	3	9

## Data Availability

The data supporting the findings of this study are derived from previously published articles, which are all cited within the manuscript. No new data were created or analyzed in this study. Data sharing is therefore not applicable.

## Supplementary Materials

The following supporting information can be downloaded at: https://www.mdpi.com/article/10.3390/jcm14238610/s1, Table S1: Biomarker assay characteristics and analytical approach in the 13 included prospective cohort studies. Table S2: PRISMA 2020 Checklist for the reporting of systematic reviews and meta-analyses.

## Abbreviations

The following abbreviations are used in this manuscript:

AHF	Acute Heart Failure
BNP	B-type Natriuretic Peptide
CI	Confidence Interval
CRP	C-Reactive Protein
CV	Cardiovascular
DAPA-HF	Dapagliflozin and Prevention of Adverse Outcomes in Heart Failure Trial
ECG	Electrocardiogram
EF	Ejection Fraction
eGFR	Estimated Glomerular Filtration Rate
ESC	European Society of Cardiology
GRADE	Grading of Recommendations, Assessment, Development and Evaluation
HF	Heart Failure
HFpEF	Heart Failure with Preserved Ejection Fraction
HFrEF	Heart Failure with Reduced Ejection Fraction
HR	Hazard Ratio
hs-CRP	High-Sensitivity C-Reactive Protein
I^2^	I-Squared Statistic (Heterogeneity)
IL-6	Interleukin-6
JAK/STAT3	Janus Kinase/Signal Transducer and Activator of Transcription 3
LVEF	Left Ventricular Ejection Fraction
MOOSE	Meta-analysis of Observational Studies in Epidemiology
NLR	Neutrophil-to-Lymphocyte Ratio
NOS	Newcastle–Ottawa Scale
NT-proBNP	N-terminal pro-B-type Natriuretic Peptide
OR	Odds Ratio
PRISMA	Preferred Reporting Items for Systematic Reviews and Meta-Analyses
PROSPERO	International Prospective Register of Systematic Reviews
RCT	Randomized Controlled Trial
SD	Standard Deviation
