# Supplementary material for: Systemic Inflammatory Biomarkers (Interleukin-6, High-Sensitivity C-Reactive Protein, and Neutrophil-to-Lymphocyte Ratio) and Prognosis in Heart Failure: A Meta-Analysis of Prospective Cohort Studies
Source: J Clin Med. 2025 Dec 4;14(23):8610. doi: 10.3390/jcm14238610 (PMC12692851; doi:10.3390/jcm14238610)
Supplement: Supplementary file 1 [file jcm-14-08610-s001.zip › jcm-4018423-supplementary.pdf]

Supplementary Table S1. Biomarker assay characteristics and analytical approach in the 13 included prospective cohort studies

**First author, year**	**Biomarker**	**Assay platform / Manufacturer**	**Units**	**Cut-off or categorisation used in the original analysis**
Markousis-Mavrogenis 2019 (BIOSTAT-CHF)	IL-6	ELISA (R&D Systems Quantikine HS)	pg/mL	Continuous (per 1-SD increase in log-transformed IL-6)
Pérez 2021 (ASCEND-HF substudy)	IL-6	Elecsys electrochemiluminescence (Roche Diagnostics)	pg/mL	Tertiles
Chia 2021 (PREVEND)	IL-6	In-house ELISA (developed at UMCG, Groningen)	pg/mL	Continuous (per log2 increase)
Berger 2024 (LURIC)	IL-6	Elecsys electrochemiluminescence (Roche Diagnostics)	pg/mL	Highest quartile (>7.8 pg/mL) vs lower quartiles
Docherty 2025 (DAPA-HF substudy)	IL-6	Olink proximity extension assay (Olink Proteomics)	pg/mL	Continuous (per doubling of IL-6)
Zhang 2023	hs-CRP	High-sensitivity immunoturbidimetric assay (Roche)	mg/L	Cumulative exposure quartiles over multiple measurements
He 2023 (China-PEACE)	hs-CRP	High-sensitivity latex-enhanced immunoturbidimetric assay	mg/L	Latent class trajectory modelling (4 trajectory groups)
Zhu 2025	hs-CRP	High-sensitivity immunoturbidimetric assay	mg/L	Highest tertile vs lower tertiles
Ferreira 2024 (TOPCAT Americas)	hs-CRP	High-sensitivity nephelometric assay (Siemens BN II)	mg/L	Continuous (per log increase) and highest quartile (>8 mg/L)
Santas 2024	hs-CRP	High-sensitivity turbidimetric immunoassay (Roche)	mg/L	>10 mg/L vs ≤10 mg/L
Turfan 2014	NLR	Automated haematology analyser (from routine CBC)	–	Highest quartile (>6.5) vs lower quartiles
Curran 2021 (BIOSTAT-CHF subanalysis)	NLR	Automated haematology analyser (routine blood count)	–	Continuous (per 1-unit increase) and >4.7 as high-risk
Davison 2022 (BLAST-AHF / RELAX-AHF pooled)	NLR	Automated haematology analyser (site-specific)	–	Highest quartile vs lower three quartiles

Supplementary Table S2: PRISMA 2020 Checklist for the reporting of systematic reviews and meta-analyses.

Section and Topic	Item #	Checklist item	Location where item is reported
**TITLE**			
Title	1	Identify the report as a systematic review.	Title page – "Systematic Review"
**ABSTRACT**			
Abstract	2	See the PRISMA 2020 for Abstracts checklist.	Structured Abstract on page 1 (follows PRISMA for Abstracts)
**INTRODUCTION**			
Rationale	3	Describe the rationale for the review in the context of existing knowledge.	Introduction, paragraphs 1–5 (context and knowledge gap)
Objectives	4	Provide an explicit statement of the objective(s) or question(s) the review addresses.	Last paragraph of the Introduction ("Accordingly, the present meta-analysis sought to…")
**METHODS**			
Eligibility criteria	5	Specify the inclusion and exclusion criteria for the review and how studies were grouped for the syntheses.	Section 2.4 – Study Selection and Eligibility Criteria
Information sources	6	Specify all databases, registers, websites, organisations, reference lists and other sources searched or consulted to identify studies. Specify the date when each source was last searched or consulted.	Section 2.3 – Literature Search Strategy (databases + last search date)
Search strategy	7	Present the full search strategies for all databases, registers and websites, including any filters and limits used.	Section 2.3 – Full database search terms and filters
Selection process	8	Specify the methods used to decide whether a study met the inclusion criteria of the review,	Section 2.4 – describes two independent

Section and Topic	Item #	Checklist item	Location where item is reported
		including how many reviewers screened each record and each report retrieved, whether they worked independently, and if applicable, details of automation tools used in the process.	reviewers and screening workflow
Data collection process	9	Specify the methods used to collect data from reports, including how many reviewers collected data from each report, whether they worked independently, any processes for obtaining or confirming data from study investigators, and if applicable, details of automation tools used in the process.	Section 2.5 – Data Extraction (two reviewers, standardized template)
Data items	10a	List and define all outcomes for which data were sought. Specify whether all results that were compatible with each outcome domain in each study were sought (e.g. for all measures, time points, analyses), and if not, the methods used to decide which results to collect.	Section 2.2 (PICO – Outcomes) and Section 2.5
	10b	List and define all other variables for which data were sought (e.g. participant and intervention characteristics, funding sources). Describe any assumptions made about any missing or unclear information.	Section 2.5 – extracted covariates and assumptions for missing data
Study risk of bias assessment	11	Specify the methods used to assess risk of bias in the included studies, including details of the tool(s) used, how many reviewers assessed each study and whether they worked independently, and if applicable, details of automation tools used in the process.	Section 2.6 – NOS methods (two reviewers, independent assessment)
Effect measures	12	Specify for each outcome the effect measure(s) (e.g. risk ratio, mean difference) used in the synthesis or presentation of results.	Section 2.7 – Statistical Analysis (HRs, OR≈HR for low events)
Synthesis methods	13a	Describe the processes used to decide which studies were eligible for each synthesis (e.g. tabulating the study intervention characteristics and comparing against the planned groups for each synthesis (item #5)).	Section 2.4 + Section 3.1 (final selection of 13 cohorts)

Section and Topic	Item #	Checklist item	Location where item is reported
	13b	Describe any methods required to prepare the data for presentation or synthesis, such as handling of missing summary statistics, or data conversions.	Section 2.7 (log transformations, OR-to-HR approximation)
	13c	Describe any methods used to tabulate or visually display results of individual studies and syntheses.	Table 1 and Figures 3–5 (forest plots)
	13d	Describe any methods used to synthesize results and provide a rationale for the choice(s). If meta-analysis was performed, describe the model(s), method(s) to identify the presence and extent of statistical heterogeneity, and software package(s) used.	Section 2.7 – random-effects model, heterogeneity, CMA v4.0 & RevMan 5.4
	13e	Describe any methods used to explore possible causes of heterogeneity among study results (e.g. subgroup analysis, meta-regression).	Section 3.5 + Figures 6–7 (subgroup analyses, meta-regression)
	13f	Describe any sensitivity analyses conducted to assess robustness of the synthesized results.	Section 3.5 (leave-one-out, exclusion of small studies)
Reporting bias assessment	14	Describe any methods used to assess risk of bias due to missing results in a synthesis (arising from reporting biases).	Section 3.3 – Egger's test; Figure 2 – funnel plots
Certainty assessment	15	Describe any methods used to assess certainty (or confidence) in the body of evidence for an outcome.	Section 2.6 (GRADE methods) and Section 3.3.1 (GRADE results)
**RESULTS**			
Study selection	16a	Describe the results of the search and selection process, from the number of records identified in the search to the number of studies included in the review, ideally using a flow diagram.	Section 3.1 + Figure 1 (PRISMA flow diagram)
	16b	Cite studies that might appear to meet the inclusion criteria, but which were excluded, and explain why they were excluded.	Section 3.1 + Figure 1 (exclusion categories)

Section and Topic	Item #	Checklist item	Location where item is reported
Study characteristics	17	Cite each included study and present its characteristics.	Section 3.2 + Table 1
Risk of bias in studies	18	Present assessments of risk of bias for each included study.	Section 3.3 + Table 2 (NOS)
Results of individual studies	19	For all outcomes, present, for each study: (a) summary statistics for each group (where appropriate) and (b) an effect estimate and its precision (e.g. confidence/credible interval), ideally using structured tables or plots.	Section 3.4 + Figures 3–5 (effect estimates and CIs for each study)
Results of syntheses	20a	For each synthesis, briefly summarise the characteristics and risk of bias among contributing studies.	Sections 3.2, 3.3, and 3.4
	20b	Present results of all statistical syntheses conducted. If meta-analysis was done, present for each the summary estimate and its precision (e.g. confidence/credible interval) and measures of statistical heterogeneity. If comparing groups, describe the direction of the effect.	Section 3.4 + forest plots (Figures 3–5) (pooled HRs, CIs, heterogeneity)
	20c	Present results of all investigations of possible causes of heterogeneity among study results.	Section 3.5 + Figures 6–7
	20d	Present results of all sensitivity analyses conducted to assess the robustness of the synthesized results.	Section 3.5
Reporting biases	21	Present assessments of risk of bias due to missing results (arising from reporting biases) for each synthesis assessed.	Section 3.3 + Figure 2
Certainty of evidence	22	Present assessments of certainty (or confidence) in the body of evidence for each outcome assessed.	Section 3.3.1 (GRADE)
**DISCUSSION**			
Discussion	23a	Provide a general interpretation of the results in the context of other evidence.	Section 4.1 – Principal findings

Section and Topic	Item #	Checklist item	Location where item is reported
	23b	Discuss any limitations of the evidence included in the review.	Section 4.4 – Strengths and Limitations
	23c	Discuss any limitations of the review processes used.	Section 4.4 (measurement heterogeneity, single-timepoint biomarkers)
	23d	Discuss implications of the results for practice, policy, and future research.	Sections 4.3 and 4.5 (clinical implications and future directions)
**OTHER INFORMATION**			
Registration and protocol	24a	Provide registration information for the review, including register name and registration number, or state that the review was not registered.	Section 2.1 – PROSPERO ID: CRD420251207035
	24b	Indicate where the review protocol can be accessed, or state that a protocol was not prepared.	Section 2.1 – protocol not publicly available
	24c	Describe and explain any amendments to information provided at registration or in the protocol.	Not applicable (no amendments)
Support	25	Describe sources of financial or non-financial support for the review, and the role of the funders or sponsors in the review.	Funding statement (Internal funding; UMFT support)
Competing interests	26	Declare any competing interests of review authors.	Conflicts of Interest section
Availability of data, code and other materials	27	Report which of the following are publicly available and where they can be found: template data collection forms; data extracted from included studies; data used for all analyses; analytic code; any other materials used in the review.	Data Availability Statement ("derived from previously published studies; no new data created")
